# Living Cellulose Materials with Tunable Viscoelasticity through Probiotic Proliferation

**DOI:** 10.1021/acsabm.2c00814

**Published:** 2022-12-15

**Authors:** Laura Sabio, Jose M. Dominguez-Vera, Juan de Vicente, José M. Delgado-López

**Affiliations:** † Department of Inorganic Chemistry, Faculty of Sciences, University of Granada, Av. Fuentenueva s/n, 18071 Granada, Spain; ‡ F2N2Lab, Magnetic Soft Matter Group and Excellence Research Unit ‘Modeling Nature’ (MNat), Department of Applied Physics, Faculty of Sciences, University of Granada, Av. Fuentenueva s/n, 18071 Granada, Spain

**Keywords:** living materials, bacterial cellulose, probiotics, viscoelasticity, stimuli-responsive materials

## Abstract

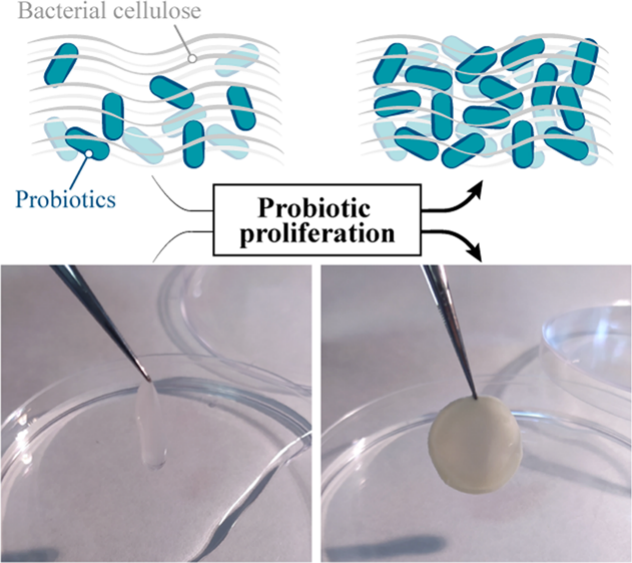

Probiotic cellulose (PC), a living material (LM) consisting of probiotics integrated into bacterial cellulose, is the first example where life (probiotic proliferation) is the input to tune the viscoelasticity of the biomaterial. The gradual proliferation of probiotics within the matrix acts as a key modulator of the cellulose viscoelasticity, providing from celluloses with lower-than-matrix viscoelasticity to celluloses with viscoelastic moduli closer to those of elastic solids. This concept is a promising approach to producing living bio-ink with tunable viscoelastic response of special interest for specific applications such as 3D printing. In contrast to the most common hydrogels with stimuli-tunable mechanical properties, which require external stimuli such as mechanical stress, UV radiation, or heat, this living bio-ink only requires time to tune from a fluid-like into a solid-like biomaterial.

## Introduction

Living materials (LMs) are an emergent class of synthetic materials formed by combining living biological entities with soft materials. In LMs, the properties of the abiotic component enhance the stability and activity of the living entity, and conversely, the living entity can either control the structure of the material counterpart or modify its properties.^1−6^

Cells in biological tissues are an example of natural LMs. Cells are living blocks surrounded by an inert extracellular matrix, which provides structural support and plays a key role in cell behavior. Synthetic LMs, mimicking cell tissues, offer then a unique opportunity for understanding how external stimuli affect the living block properties, an ongoing situation in biological systems. In addition, LMs are unique examples of stimuli-responsive materials (SRM)^7−10^ as they can react to external stimuli through both the living and nonliving blocks. LMs have the potential to undergo a cascade process of alteration of their structure and functions after exposure to an external signal that provokes, first, a response of the living block, which subsequently affects the inert matrix, concluding in definitive changes on the structure and properties of the LM.

The stimulus–response behavior in most of the SRM is typically triggered by external stimuli such as light, temperature, chemicals, or electric fields.^11−15^ However, very few examples have been described where an internal stimulus comes from the material environment itself, a scenario taking place in cells and biological tissues.

In this context, we present here a new LM, the so-called probiotic cellulose (PC),^16^ consisting of a nonliving matrix of bacterial cellulose (BC) in which living and active probiotics are perfectly integrated. In PC, the incubation time produces, first, an increase in cell proliferation, which gradually tunes the viscoelastic response of the cellulose matrix, *i.e.*, resulting from celluloses with lower-than-matrix viscoelasticity (at a low probiotic density) to celluloses with viscoelastic moduli close to those of elastic solids (at a higher probiotic density). We can, therefore, stimulate bacterial proliferation and control the viscoelasticity of PC.

Viscoelasticity is the tendency of a material to act both like an elastic solid and a viscous liquid depending on the ratio between the characteristic time of relaxation of the material and the time of observation (*i.e.*, the so-called Deborah number^17^). Viscoelastic materials show intermediate behavior between a Newtonian (linear viscous) liquid and a Hookean (elastic) solid.^18^

Viscoelasticity is relevant for a broad range of applications and plays a key role in many processes ranging from the extrusion of polymer melts into molds to the bread-making performance of dough. Viscoelasticity is also crucial in natural biological materials, such as cartilage and skin,^19−21^ and synthetic ones, such as shaving foams and paints. Bone, for instance, is a good example of viscoelastic material that exhibits creep deformation and stress relaxation.^22^ Thus, novel living materials with tunable viscoelasticity, such as probiotic cellulose, represent lab-made systems to study the influence of the living entities, in particular cell proliferation, on the mechanical response of the biological material. Moreover, these responsive living materials could be used as bio-inks to obtain health devices, probiotic cellulose being of special interest due to its antimicrobial properties against even antibiotic-resistant bacteria, as recently shown.^16^

## Materials and Methods

### Reagents and Solutions

High-grade quality reagents were used as received from commercial suppliers unless otherwise stated. Aqueous solutions were prepared with ultrapure water (18.2 MΩ·cm, bacteria: <0.1 CFU/mL at 25 °C, Milli-Q, Millipore).

### Bacteria Culture

Certain types of bacteria, those classified as acetic acid bacteria being especially relevant, are able to produce bacterial cellulose (BC). Within this group, *Komagataeibacter xylinus* (*Kx*) is widely used since it shows a high yield of BC synthesis from a broad range of nitrogen and carbon sources, and thus, it is considered one of the most effective.^23^ The lyophilized *Kx* (ATCC 11142, CECT 473) was supplied by the Colección Española de Cultivos Tipo (CECT) and grown in Hestrin–Schramm (HS) agar^24^ at 30 °C. The HS medium formula is (w/v) 2% glucose, 0.5% yeast extract, 0.5% peptone, 0.115% citric acid, and 0.68% Na_2_HPO_4_·12H_2_O (Sigma). *Lactobacillus fermentum* (*Lf*) was kindly provided by Biosearch Life S.A. and grown in de Man, Rogosa, and Sharpe medium (MRS, Oxoid) at 37 °C.

### Bacterial Cellulose Synthesis and Incorporation of Probiotics by Co-culture

**Figure 1:**
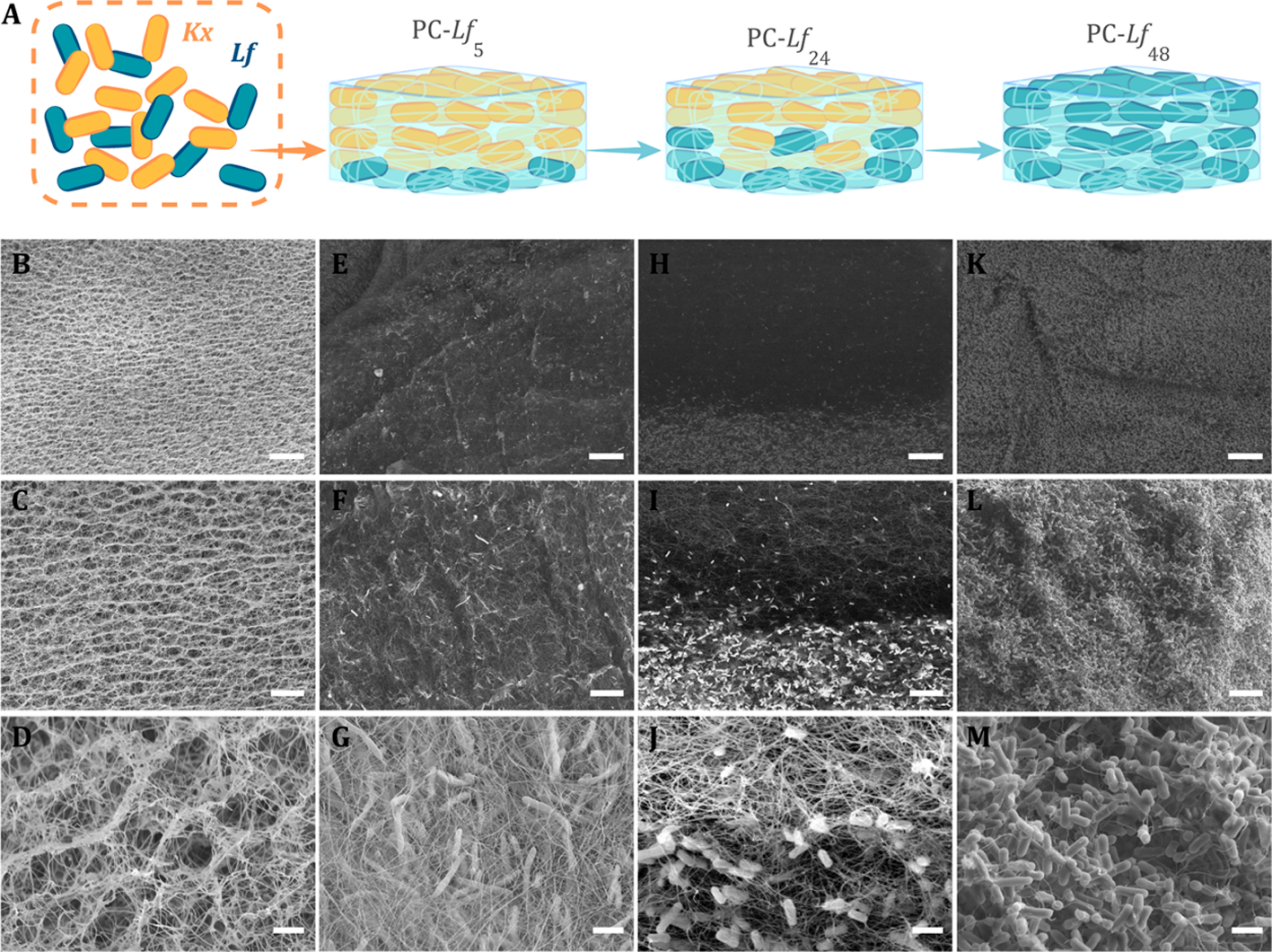
(A) Graphical representation of the experimental protocol used to build PC-*Lf* through the co-incubation of *Kx* and *Lf*. Cross-sectional FESEM images of (B–D) BC, (E–G) PC-*Lf*_5_, (H–J) PC-*Lf*_24_, and (K–M) PC-*Lf*_48_ at increasing resolutions (from top to bottom). Scale bars = 20, 10, and 2 μm (from lower to higher magnifications, *i.e.*, from top to bottom).

The LM, consisting in probiotics integrated in bacterial celluloses (hereafter referred to as PC-*Lf*) were synthesized through a previously reported protocol.^16^ Briefly, 0.1 mL of *Kx* suspension (OD_600nm_ = 0.3) was mixed with 0.1 mL of *Lf* suspension (OD_600nm_ = 0.3) in 1 mL of HS medium and co-cultured in aerobic conditions at 30 °C for 3 days. This produces a thick gel-like cellulose membrane at the liquid–air interface containing the *Kx* and *Lf* bacteria. This material was referred to as PC-*Lf*_0_. Afterward, HS medium was replaced with MRS and PC-*Lf*_0_ was incubated in anaerobic conditions at 37 °C for 5, 24, and 48 h (samples referred to as PC-*Lf*_5_, PC-*Lf*_24_, and PC-*Lf*_48_, respectively). The MRS broth was replaced after 24 h. A scheme of this protocol is depicted in Figure 1A.

Some samples were also prepared as controls. First, bacterial cellulose (without probiotics) was produced out by culturing 0.1 mL of *Kx* suspension (OD_600nm_ = 0.3) in 1 mL of HS broth and aerobic conditions at 30 °C for 3 days. After incubation, a thick gel-like membrane was produced in the liquid–air interface, composed of *Kx* bacteria and cellulose. The membranes were then immersed in ethanol 96° for 15 min followed by immersion in boiling water for 40 min and four washings of 0.1 M NaOH at 90 °C of 20 min each. Finally, the pure cellulosic pellicles were washed with distilled water until neutral pH was achieved. This purification treatment eliminates *Kx*, giving rise to pure bacterial cellulose (hereafter referred to as BC).^25^

Subsequently, a set of samples were produced by the so-called adsorption–incubation method.^26^ BC was completely immersed in MRS media containing the probiotics. The same incubation times (5, 24, and 48 h) were used, and the resulting materials were referred to as BC + *Lf*_5_, BC + *Lf*_24_, and BC + *Lf*_48_. A scheme of this protocol is depicted in Figure S7A (Supporting Information).

The synthesis of the materials was carried out in 2 cm-diameter vials to obtain circular samples with appropriate dimensions to carry out the rheological experiments. All the samples presented the same thickness (*ca*. 1.5 ± 0.2 mm) as measured during the rheological experiments under compression (first interval, vide infra).

### Rheological Experiments

A torsional rheometer (MCR302 Anton Paar) was used to investigate the mechanical properties of cellulose matrices both in compression and shearing mode. Experiments for BC, PC-*Lf*, and BC + *Lf* samples were carried out at 25 °C in a parallel plate configuration (20 mm diameter). We used plates with rough surfaces to prevent sample slippage. The samples were kept immersed in sterile saline solution (NaCl 0.9% w/v) before testing to avoid water loss and bacterial swelling.

Rheological experiments were performed for each type of sample in triplicate (*i.e.*, three different membranes were synthesized for each incubation time) in two different intervals:

#### Compression Test (Interval 1)

The sample was placed on the bottom plate with the help of forceps and kept immersed in saline solution throughout the experiment. The upper plate was displaced downward (*i.e.*, closing the gap) at a constant velocity (υ = 10 μm/s). During the plate motion, the normal force acting on it, due to the sample, was monitored. The upper plate stopped when the normal force (*F_N_*) reached a value of 0.3 N. This extraordinarily low force, which is distributed on the plate of the rheometer generating a pressure of only 0.6 kPa, does not affect bacteria integrity.

#### Shearing Test (Interval 2)

During this interval, the sample was confined between the two plates at a constant normal force of *F_N_* = 0.3 N and subjected to an oscillatory strain of increasing strain amplitude at a constant frequency (*f* = 1 Hz).

Using this particular protocol, it is possible to investigate both the compressive and shearing properties of the same sample. From the first interval, it is possible to obtain the compressive modulus. From the second interval, it is possible to elucidate the storage and loss moduli in the viscoelastic linear region as well as the onset of nonlinearity under shear.

### Viscosity of *Lf* Suspensions

We also analyzed *Lf* suspensions at different concentrations in sterile saline solution 0.9% (w/v). A volume of 0.7 mL of different bacterial concentrations were used, ranging from 10^10^ colony forming units (CFU)/mL to 5 × 10^4^ CFU/mL. The experiments were performed in a cone-plate geometry (50 mm diameter and 1° angle) in three intervals by triplicate. The first interval consists in a preshear to remove the mechanical history of the sample (shear rate of 500 s^–1^). In the second interval, the sample is allowed to rest for a short time. In the third interval, the sample is sheared at an increasing shear rate from 0.01 to 1 s^–1^.

### Bacterial Viability Tests

Bacterial viability and distribution of PC-*Lf*_0_, PC-*Lf*_5_, and PC-*Lf*_48_ were qualitatively assessed by confocal laser scanning microscopy (CLSM). The samples were washed with sterile saline solution and stained with a live/dead BacLight Bacterial Viability Kit (ThermoFisher) following manufacturer’s instructions. This assay combines membrane-impermeable DNA-binding stain, *i.e*., propidium iodide (PI), with membrane-permeable DNA-binding counterstain, SYTO9, to stain dead and live bacteria, respectively. Cell viability along the BC matrix was evaluated with a confocal microscope (Nikon Eclipse Ti-E A1, Centre for Scientific Instrumentation, University of Granada, CIC-UGR) equipped with a 20× objective. For acquiring SYTO9 signals (green channel), a 488 nm laser and 505–550 nm emission filter were used. For PI (red channel), a 561 nm laser and 575 nm long-pass emission filter were used. Images were analyzed with NIS Elements software.

### Field-Emission Scanning Electron Microscopy (FESEM)

BC and PC-*Lf* samples were transversally cut and fixed in 1 mL of cacodylate buffer (0.1 M, pH 7.4) containing 2.5% of glutaraldehyde at 4 °C for 24 h. Subsequently, samples were washed with cacodylate buffer three times for 30 min at 4 °C. The samples were stained with osmium tetroxide (OsO_4_) solution (1% v/v) for 2 h in the dark and then repeatedly rinsed with Milli-Q water to remove the excess of OsO_4_ solution. Samples were then dehydrated at room temperature with ethanol/water mixtures of 50, 70, 90, and 100% (v/v) for 20 min each, the last concentration being repeated three times, and dried at the CO_2_ critical point. Finally, dehydrated samples were mounted on aluminum stubs using carbon tape, sputtered with a thin carbon film, and analyzed using a FESEM (Zeiss SUPRA40V) of the CIC-UGR. The cross sections and surfaces of BC, PC-*Lf*_5_, PC-*Lf*_24_, and PC-*Lf*_48_ were analyzed.

### Gram Staining of the PC-*Lf*_48_ Sample

PC-*Lf*_48_ was dehydrated in gradient ethanol and washed with xylene. The sample was then embedded in paraffin and transversally cut in 4 μm sections using a microtome. Slides were deparaffinized, cleared in xylene, and rehydrated. Then, a standard Gram staining protocol was performed to differentiate between the Gram-negative cellulose-producing bacteria, *Kx*, and the Gram-positive probiotic, *Lf*. In brief, crystal violet was applied for 1 min at room temperature, and slides were briefly rinsed under running water to remove the excess of staining. Iodine mordant was applied for 30 s and washed with water. Slides were covered with ethanol for 15 s and quickly rinsed under running water until the water run clear. Finally, safranin was applied for 1 min and rinsed with water. The slides were observed using an iScope (Euromex) microscope in bright field mode and under a 100× immersion oil objective.

### Estimation of *Kx*/*Lf* Fractions

Samples were first digested with cellulase from *Trichoderma reesei* (no. C2730-50ML, Sigma-Aldrich).^16^ Each sample was incubated in 3 mL of enzyme solution (50 μL of cellulase/mL of potassium phosphate buffer, 50 mM, pH 6) at 37 °C and 180 rpm for 1 h. The aliquots of the bacterial suspension were also fixed on glass slides and stained according to the Gram staining procedure described before. To estimate the *Kx*/*Lf* bacterial fraction of each sample, Gram-negative and Gram-positive bacteria from different areas of the slide were counted (*n* = 200).

### Cellulose Fiber Width Distribution

Fiber widths (*n* = 100) of BC, PC-*Lf*_5_, and PC-*Lf*_48_ were measured from FESEM images using ImageJ 1.52a software (http://imagej.nih.gov/ij).

### X-ray Diffraction (XRD) Patterns

X-ray diffraction (XRD) patterns were collected from the surface of lyophilized cellulose films using a Bruker D8 Discover diffractometer (Centre of Scientific Instrumentation, University of Granada) equipped with a 2D detector (Pilatus3R 100K-A, Dectris) at 25 °C with Cu Kα (λ = 1.5406 Å) radiation generated at 50 kV and 1 mA. The XRD diffraction patterns were recorded at a rate of 40 s/step from 10 to 40° with a step size of 0.02°. Each spectrum was baseline-corrected and normalized to the maximum intensity at a 2θ value of around 22.5°.

Freeze-drying of the samples was carried out by freezing in liquid nitrogen for 10 min before drying under vacuum at −60 °C for 2 days using a Telstar Cryodos-50.

### Statistics and Graphs

Results were analyzed with the software OriginPro 8 and Excel, and data were expressed as mean of *n* = 3 (three samples measured for each condition) ± standard deviations. Statistical analysis was performed with GraphPad Prism v5.0, and the software MagicPlot Pro 2.9.3 was used for data plotting.

## Results and Discussion

### Synthesis and Characterization of LMs

The co-cultivation of aerobic *Kx* and facultative anaerobic probiotics (such as *Lf*) in HS media under aerobic conditions results in the formation of a cellulose film containing both *Kx* and *Lf* (Figure 1A). Interestingly, the incubation of this film in the optimal conditions for the probiotic (anaerobic environment and MRS medium) causes the proliferation of *Lf*, which gradually invades the whole cellulose matrix (Figure S1E–H). Figure 1B–M shows the cross-sectional FESEM images of PC-*Lf* samples obtained at 5, 24, and 48 h of incubation in anaerobic MRS media (referred to as PC-*Lf*_5_, PC-*Lf*_24_, and PC-*Lf*_48_, respectively) and pure BC for the sake of comparison. At short incubation times, the cellulose contained mainly fibrous-like *Kx* bacteria but some probiotics can be observed proliferating from the bottom (Figure 1G and Figure S2D). The number of probiotics increases at 24 h until they invade the whole matrix after 48 h (Figure 1K–M). In fact, the FESEM images of the two opposite surfaces of PC-*Lf*_48_ appeared fully covered of *Lf* (Figure S2C,F). Additionally, PC-*Lf*_48_ was stained by crystal violet/safranin (Gram stain), and all the cells resulted stained in purple, confirming that the whole pellicle was invaded by the Gram-positive probiotics (Figure S2G).

Samples were also degraded with cellulase to extract the cells, which were then analyzed by optical microscopy and Gram staining (Figure S1). Two types of morphologies were observed: long fibrous Gram-negative *Kx* (which were stained pink by safranin) and rod-like Gram-positive *Lf* (appearing in purple due to crystal violet) (Figure S1E–H). In agreement with FESEM observations, the percentage of *Kx* decreases with the proliferation time under anaerobic conditions, from 93% (PC-*Lf*_0_) to less than 0.5% (PC-*Lf*_048_) (Figure S1).

In contrast to that produced by plants, BC forms long fibers with nanometric diameters (10–80 nm), which confers it with a very high specific surface area, water-holding property, and absorption capability.^27^ The diameter of the cellulose fibers (60 nm average, Figure S3A) was similar in all the samples, and the corresponding XRD patterns (Figure S3B), with the characteristic diffraction peaks of cellulose assembled nanocrystals, neither showed noticeable structural differences (despite the increase in diffuse scattering coming from the increasing bacteria density).^28^ These results confirmed that the presence of probiotics did not affect the microstructure of the cellulose network.

**Figure 2:**
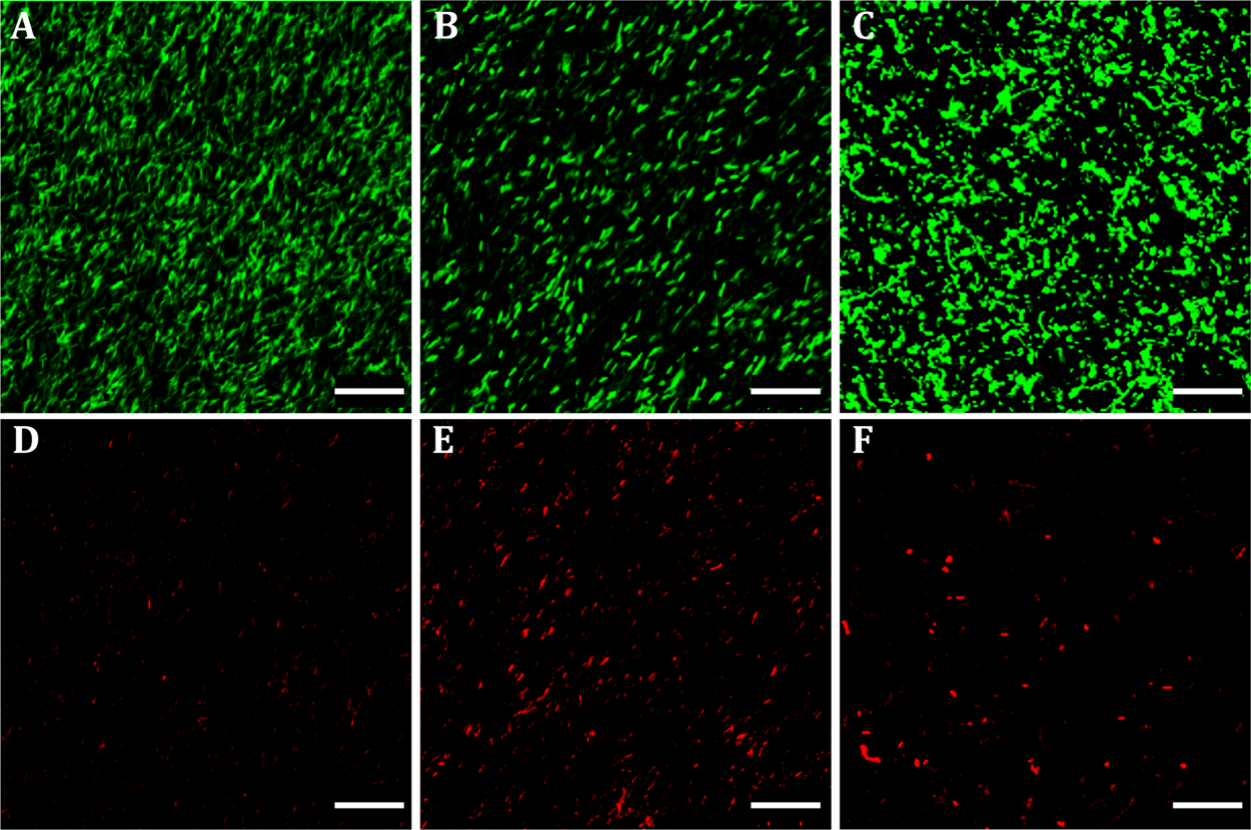
CLSM images of stained (A, D) PC-*Lf*_0_, (B, E) PC-*Lf*_5_, and (C, F) PC-*Lf*_48_. (A–C) Green channel (SYTO 9), (D–F) Red channel (propidium iodide). Scale bars = 20 μm.

Figure 2 shows the confocal laser scanning microscopy (CLSM) images of PC-*Lf* samples labeled with the pair SYTO9/propidium iodide fluorescent dyes (live/dead assays). Irrespective of the proliferation time, most of the bacteria were stained in green but not in red, indicating that they were alive. The number of red-labeled (dead) bacteria increases for the samples cultured under anaerobic conditions (PC-*Lf*_5_ and PC-*Lf*_48_). This progressive death of the aerobic *Kx* was caused by the anaerobic conditions used during probiotic proliferation. These images also confirmed that the bacterial density depends on the cultivation time.

### Mechanical Response of the LMs

The mechanical response of the PC-*Lf* biomaterials was initially assessed by compression tests. Cellulosic samples were progressively squeezed at constant velocity until a normal force (*F_N_*) of 0.3 N (954.93 Pa) was reached. The experimental setup and raw data of *F_N_* as a function of the gap separation (*h*) are presented in Figure S4. PC-*Lf* samples as well as BC exhibited a two-step response. At large initial gaps, water was gradually expelled from the samples so the normal force increases slowly when closing the gap. At the end of the compression process (small gaps), the plate was inelastically deforming the cellulose matrix so that the normal force increases quicker when decreasing the gap separation. The compression moduli *E*, *i.e.*, the slope of the stress (σ) vs strain (ε) curves, were calculated in the range 0 < ε < 0.3 (Figure S4D). The compression modulus accounts for the resistance of the material to be compressed. As observed, the compression modulus in the low strain region was similar for all the samples (Figure S4E) and was in agreement with the moduli of cellulosic samples measured under similar conditions.^29^ This result was expected because, as discussed before, the structure of the cellulose fibrillar network apparently was not affected by the presence of the different bacteria over culture time. However, the situation at higher strains is rather different. Samples with a high density of probiotics (PC-*Lf*_48_) were inelastically deformed at a much lower strain than the matrix BC (Figure S4D). On the contrary, higher strains were needed to inelastically deform the samples with a low density of probiotics (PC-*Lf*_0_, PC-*Lf*_5_, and PC-*Lf*_24_), with the sample PC-*Lf*_5_ being inelastically deformed at strains close to 1 (Figure S4D).

We also measured the gap separation between the plates of the rheometer both when the plates get in contact with the sample (*h*_0_, when *F_N_* starts to increase) and at the end of the compression (*h*_1_, when *F_N_* = 0.3 N). The results are plotted in Figure S5. All the samples presented practically the same thickness with *h*_0_ values of *ca*. 1.5 mm. However, *h*_1_ was highly dependent on the amount of probiotics (Figure S5). PC-*Lf*_48_ showed an *h*_1_ significantly longer than BC. This is a consequence of the high density of *Lf* inside the cellulose backbone. On the contrary, samples with a low probiotic density (PC-*Lf*_0_ and PC-*Lf*_5_) showed an unusually high compressibility. In fact, the final gap *h*_1_ of these samples was around 10 times thinner than that of the BC matrix (Figure S5B). All these observations confirmed that the bacterial density within the matrix plays a key role in the mechanical response of the biomaterials.

**Figure 3:**
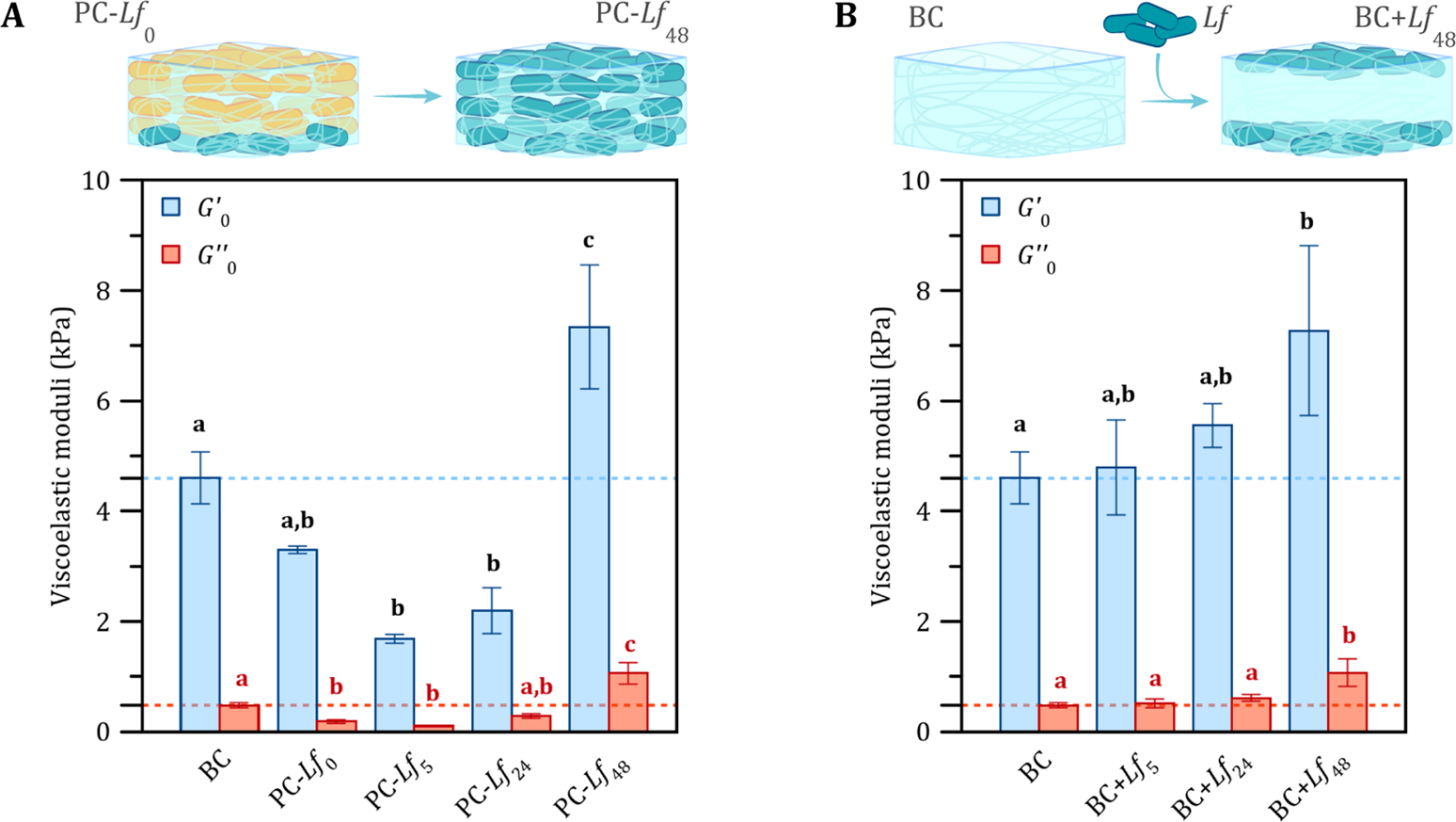
Viscoelastic moduli (*G*_0_^′^, *G*_0_^″^) in the linear viscoelastic region (LVR) as obtained from strain amplitude sweep tests for (A) PC-*Lf* and for (B) BC + *Lf* (obtained by the adsorption–incubation method) with increasing contents of bacteria. Moduli of pure BC are also shown for comparison. Statistical analysis was carried out by one-way ANOVA (Bonferroni’s method). Different letters indicate significant differences (*p* < 0.05) between samples (black and red letters for *G*_0_^′^ and *G*_0_^″^, respectively).

Once the samples were confined between the plates (*F_N_* = 0.3 N), dynamic oscillatory shear tests were carried out by increasing the strain amplitude from 10^–4^ to 200% at a constant frequency of 1 Hz to explore their viscoelastic characteristics under shear. The experimental setup and the resulting averaged curves are shown in Figure S6. For sufficiently small strains, both viscoelastic moduli remain flat (this is the so-called linear viscoelastic region, LVR). Here, the storage modulus (*G*′) is greater than the loss modulus (*G*″), indicating an elastic behavior. The plateau values of these linear regions (*G*_0_^′^, *G*_0_^″^) are shown in Figure 3A. When the strain amplitude increases, *G*′ and *G*″ decrease, indicating the onset of nonlinearity (linearity limit or yield point, Figure S6B). For strains above the flow point (*i.e*., *G*′ = *G*″), the sample dissipates more energy than it can store.

Substantial differences are found for *G*_0_^′^ and *G*_0_^″^, both moduli being highly dependent on bacterial density. The growth of probiotics produces a huge impact on the viscoelastic moduli. The viscoelastic moduli of PC-*Lf* samples first decrease with incubation time until a minimum is reached and then start to increase (Figure 3A). The probiotic growth inside the cellulose matrix provides materials with lower viscoelastic moduli than the matrix BC (*i.e.*, lower-than-matrix viscoelasticity), which is an unconventional situation. Subsequently, this trend changes and the moduli increase with incubation time (probiotic amount). Thus, this allows obtaining cellulose matrices with lower o higher viscoelastic moduli than the matrix itself by bacterial filling.

The viscoelasticity of these materials seems to be the result of a compromise between two factors: a small bacterial fraction favors sliding between cellulose fibers by the so-called ball-bearing effect,^30,31^ while a large fraction hinders such sliding. In the context of PC samples, the entrapped bacteria in the cellulose network can be considered sliding balls. A reduced bacterial density favors the sliding between fibers. However, this ball-bearing effect is lost above a certain number of bacteria. In this sense, it should be noted that a similar correlation exists between dislocation density and the hardness of a metallic material. At low dislocation density, plastic deformation occurs when dislocations move. However, high dislocation density hinders the motion, which results in a harder material.^32^

Interestingly, this density-dependent ball-bearing effect was only observed when the bacteria were perfectly integrated into the matrix and a real hybrid material was at work (PC-*Lf* samples). In fact, we have prepared a set of celluloses containing *Lf* probiotics by the adsorption–incubation method^26^ (BC + *Lf*, described in the Materials and Methods Section). This method produces probiotic-containing celluloses where probiotics (*Lf*) are exclusively adsorbed onto the external surfaces of BC since the dense cellulose fibril network does not allow the penetration (Figure S7). When probiotics are exclusively located at the surface, such a ball-bearing effect was not observed (Figure 3B). Thus, in contrast to PC-*Lf* samples, the viscoelastic moduli of BC + *Lf* samples increased almost linearly with the incubation time (bacterial proliferation), being greater than that of the BC matrix, irrespective of the bacterial density (Figure 3B). A similar trend was observed when the bacteria proliferated free in aqueous media (Figure S8). In this case, the viscosity of the bacterial suspension increased with bacteria concentration as typically occurring in colloidal dispersions.

**Figure 4:**
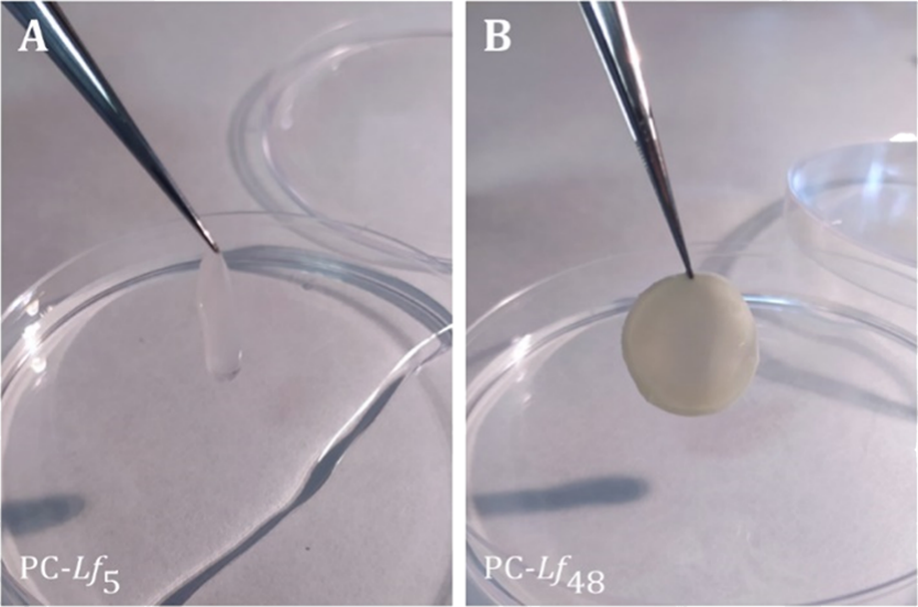
Photographs of (A) PC-*Lf*_5_ and (B) PC-*Lf*_48_. The diameter of samples was 2 cm.

Figure 4 illustrates the impact of the change of viscoelasticity in the living PC-*Lf*. PC-*Lf*_5_, the sample having the lowest viscoelastic moduli, is a transparent gel-like fluid pellicle, whereas PC-*Lf*_48_, the sample with the highest moduli, looks like an opaque solid due to the high density of probiotics inside.

## Conclusions

The viscoelasticity of probiotic cellulose, a living material consisting of probiotics integrated in bacterial cellulose, can be tuned with probiotic growth. Indeed, living materials with lower-than-matrix viscoelasticity, obtained at a low probiotic density, become an elastic solid by probiotic proliferation. This is an innovative concept of living materials, where life itself (probiotic proliferation), and no external stimuli such as UV radiation, heat, etc., makes the transformation from a relatively fluid material to elastic solid. The results here presented open the door to generate a new class of responsive living materials that could be used as bio-inks to obtain, by 3D printing, health devices. This is especially relevant for probiotic celluloses, considering that these living materials have antimicrobial properties, being even effective against antibiotic-resistant bacteria.^16^

## Supporting Information Available

Figures S1–S8 showing additional experimental details and FESEM, CLSM, and optical microscopy images; XRD patterns; and raw data derived from rheological experiments (PDF)
