# Supplementary material for: Living Cellulose Materials with Tunable Viscoelasticity through Probiotic Proliferation
Source: ACS Appl Bio Mater. 2022 Dec 15;6(1):157–63. doi: 10.1021/acsabm.2c00814 (PMC9846690; doi:10.1021/acsabm.2c00814)
Supplement: Supplementary file 1 — mt2c00814_si_001.pdf [file mt2c00814_si_001.pdf]

Supporting Information

Living Cellulose Materials with tunable viscoelasticity through probiotic proliferation

Laura Sabio,[a] Jose M. Dominguez-Vera*,[a], Juan de Vicente*,[b] and José M. Delgado-López*,[a]

[a]Department of Inorganic Chemistry, Faculty of Sciences, University of Granada, Av. Fuentenueva s/n. 18071 Granada, Spain

[b]F2N2Lab, Magnetic Soft Matter Group and Excellence Research Unit 'Modeling Nature' (MNat), Department of Applied Physics, Faculty of Sciences, University of Granada, Av. Fuentenueva s/n, 18071, Granada, Spain.

(*) Authors whom correspondence should be addressed to: J.M.D-V. (josema@ugr.es); J.d.V (jvicente@ugr.es); J.M.D.-L. (jmdl@ugr.es).

Table of Contents:

Supporting figures

- **Figure S1.**
- **Figure S2.**
- **Figure S3.**
- **Figure S4.**
- **Figure S5.**
- **Figure S6.**
- **Figure S7.**
- **Figure S8.**

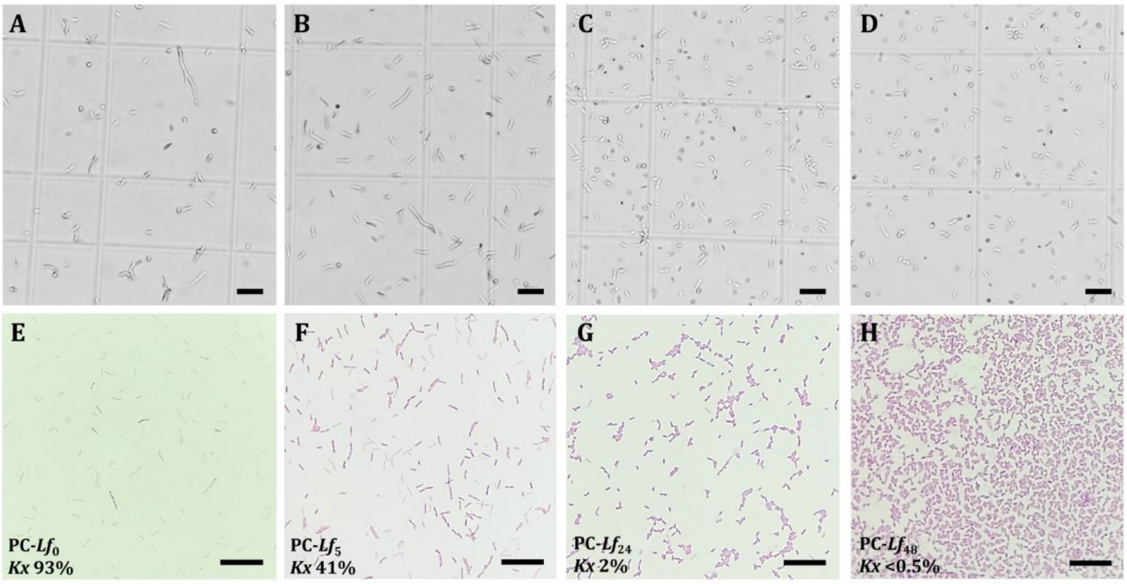

Figure S1. **(A-D)** Bright field images of bacterial suspensions after enzymatic digestion of PC-*Lf*$_0$, PC-*Lf*$_5$, PC-*Lf*$_{24}$, and PC-*Lf*$_{48}$ using the Neubauer chamber (**A-D**, respectively), and **(E-H)** after Gram staining procedure. Scale bars = 10 µm (A-D) and 20 µm (E-H). The percentages indicate the percentage of *Kx* of each sample.

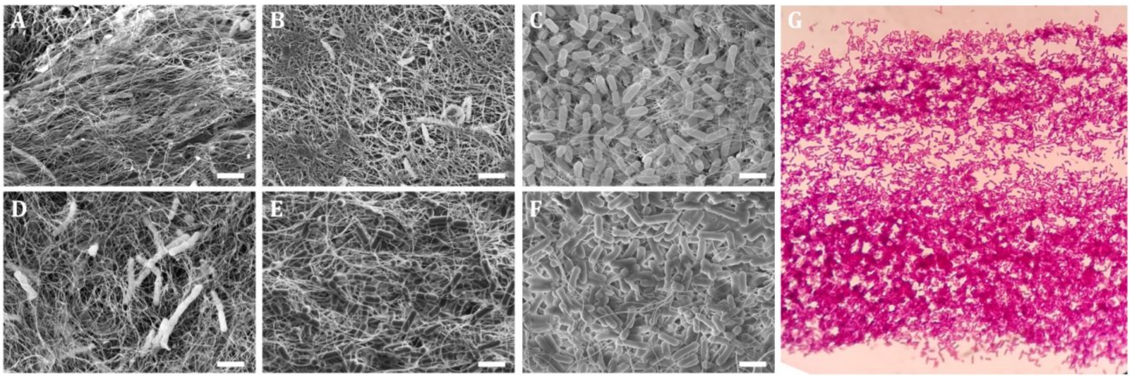

Figure S2. FESEM images of both surfaces of PC-*Lf*$_0$, PC-*Lf*$_5$ and PC-*Lf*$_{48}$ (**A-C** correspond to the surface in contact with air during incubation, and **D-F** correspond to surface in contact with the solution, respectively). Scale bars = 2 µm. (**G**) Optical microscopy of a cross-section of PC-*Lf*$_{48}$ after Gram staining (100x immersion objective). These results corroborate that both sides of PC-*Lf*$_{48}$ membranes are similar as a consequence of probiotic proliferation from one side (solution) to the other (air).

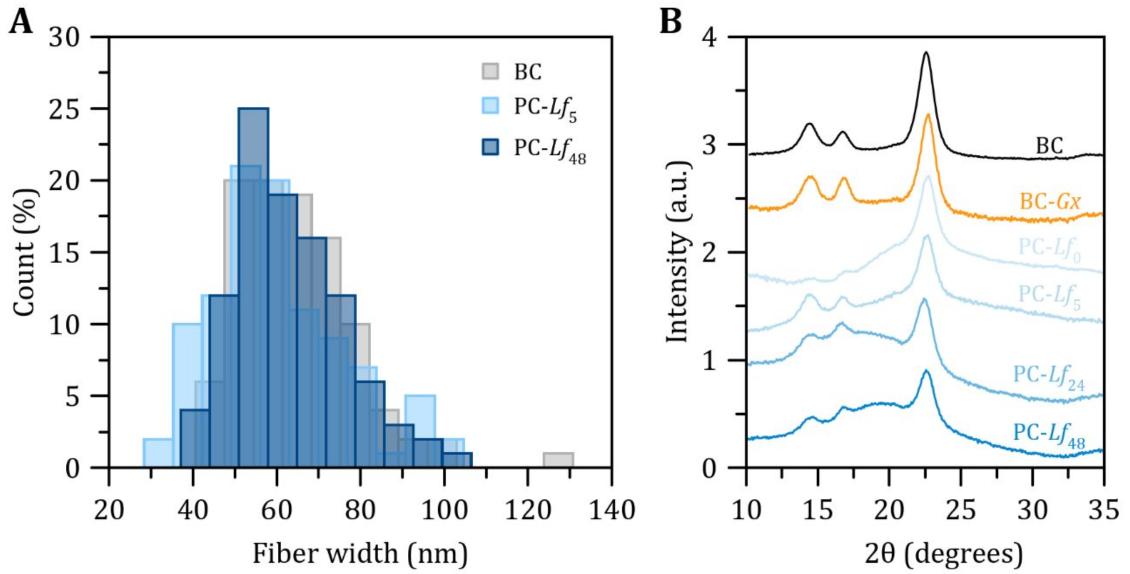

Figure S3. (**A**) Histogram of the fiber widths of BC, PC-Lf_5 and PC-Lf_{48}. As obtained from FESEM micrographs (n =100). (**B**) X-ray diffraction patterns of BC, BC-*Kx* and PC with different probiotic densities.

.

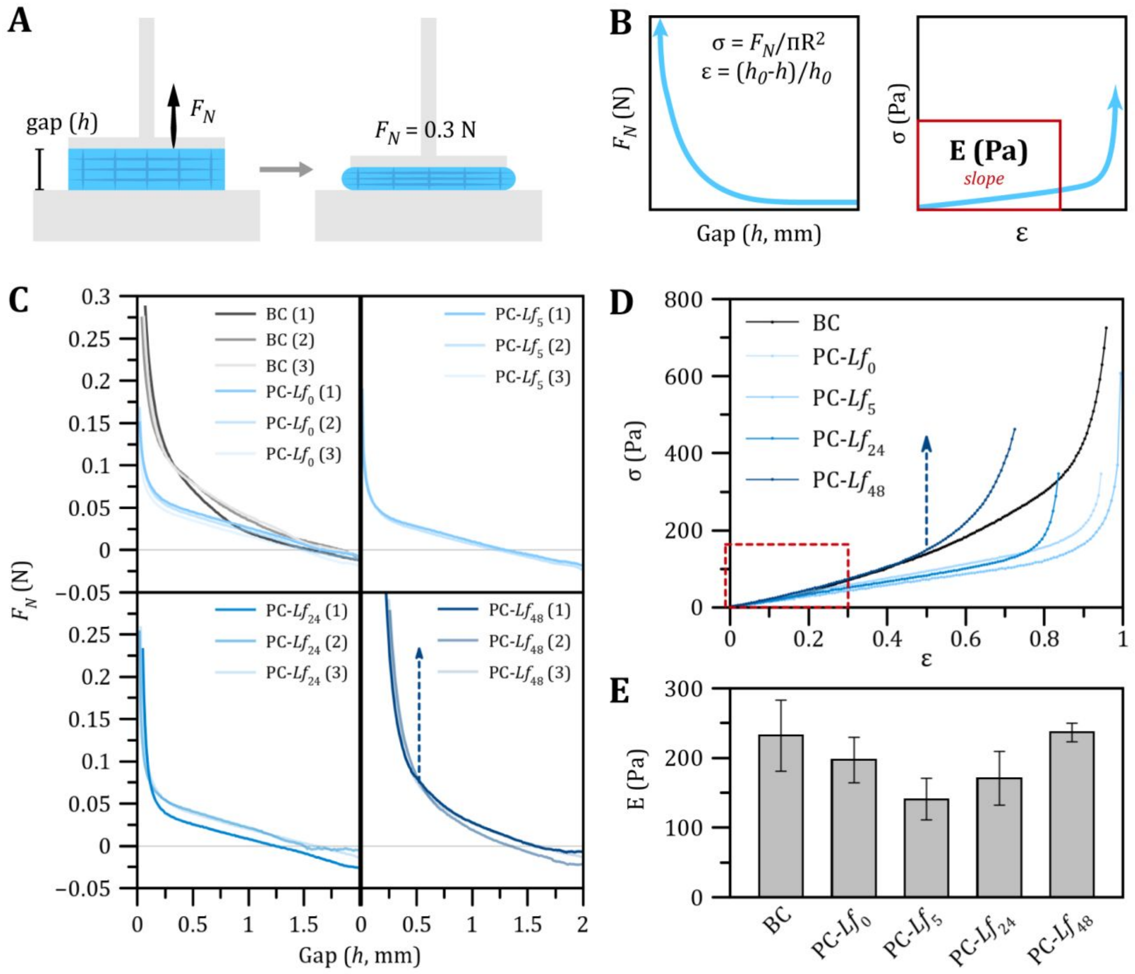

Figure S4. (**A**) Scheme of the experimental setup used during the compression tests. (**B**) Graphical representation of the extracted information. (**C**) Plots of the normal force (F_N) as a function of the gap separation distance (h) measured during compression tests of BC and PC. Each measurement was performed in triplicate (1-3) to confirm the good reproducibility between experiments with different samples prepared under the same experimental conditions. Compression curves are typical of samples with a two-step response. The blue arrow represents the force at which the plate starts to deform inelastically the cellulosic sample. (**D**) Plots of the compressive stress ($\sigma = F_N/\pi r^2$, being r the radius of the plate, 1 cm) *vs* strain ($\varepsilon = (h_0 - h)/h_0$, being h_0 the gap separation when the plate contacts the sample, $F_N > 0$). Data errors are lower than 11 Pa (in the first-step response). The slope of the interval $\varepsilon \in [0,0.3]$ is estimated as the compression modulus E, represented in panel (**E**) as vertical bars. Non-significant differences were found for E values ($p < 0.05$) after performing one-way ANOVA, Bonferroni's post-test.

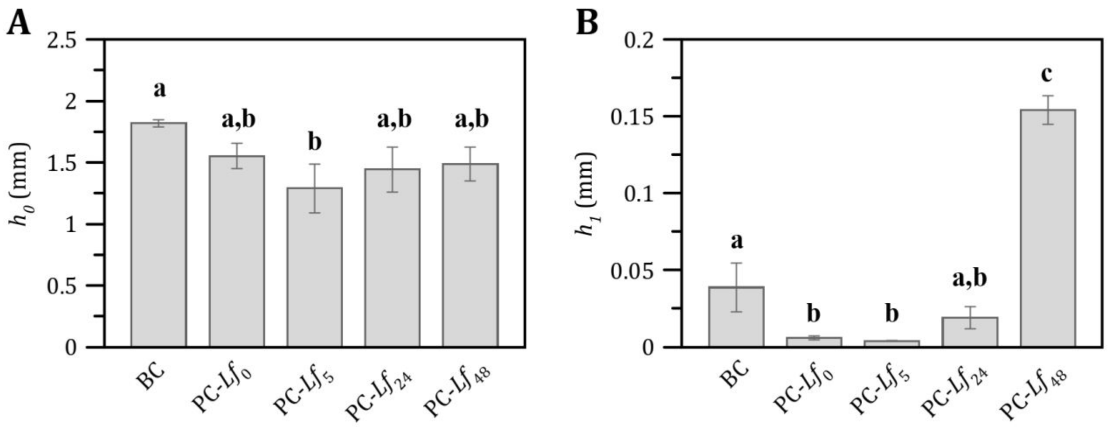

Figure S5. Gap separation between the plates of the rheometer (h) when the upper plate is set in motion and contacts the sample (**A**, $h = h_0$), and after a normal force of 0.3 N is achieved (**B**, $h = h_1$). Letters indicate significant differences ($p < 0.05$) between samples after one-way ANOVA, Bonferroni's method analysis.

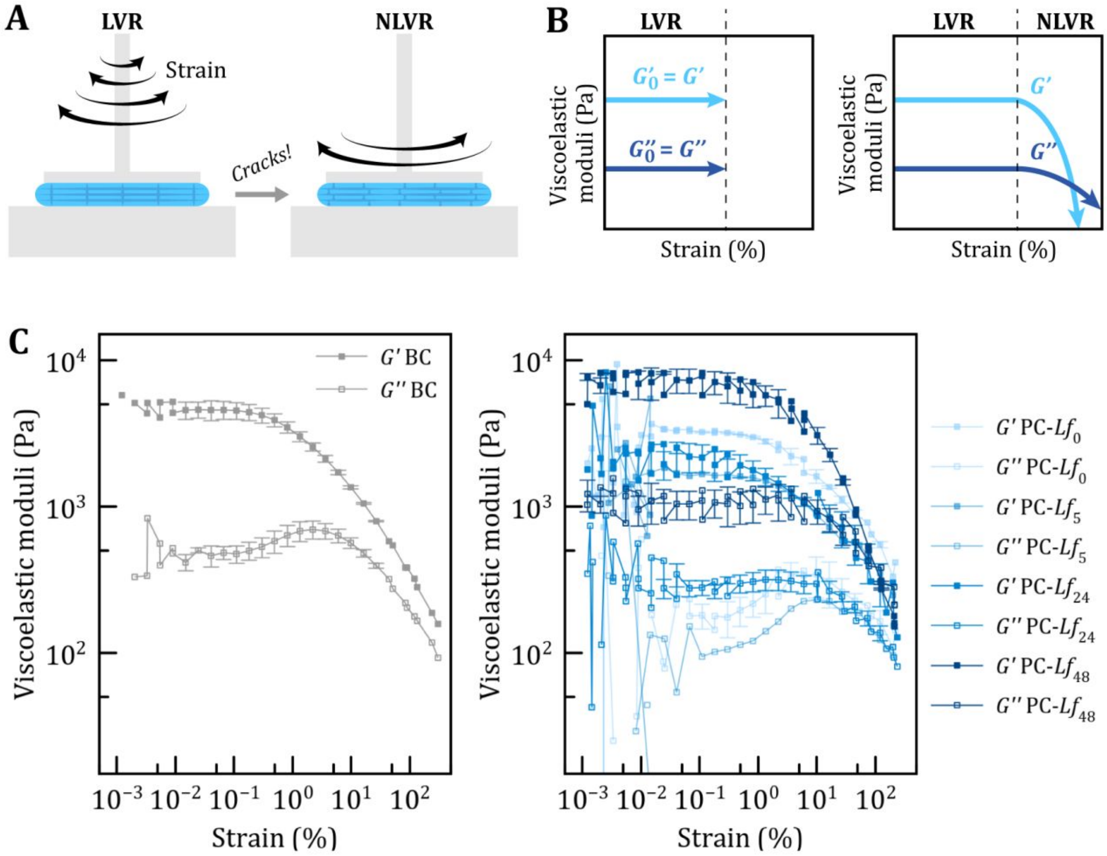

Figure S6. (**A**) Scheme of the experimental setup used during the strain-amplitude sweep tests. (**B**) Graphical representation of the extracted information. (**C**) Averaged data of strain amplitude sweep tests of BC (left), and PC-Lf_0, PC-Lf_5, PC-Lf_{24}, and PC-Lf_{48} (right). Data show means ± SD of triplicates.

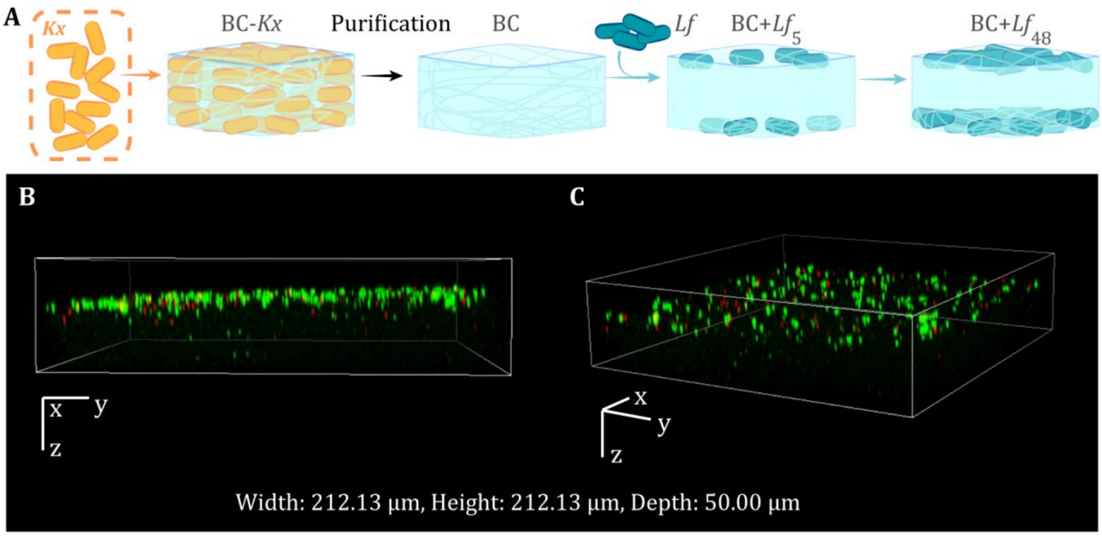

Figure S7. (**A**) Graphical representation of the experimental procedure to obtain BC with adsorbed probiotics through the adsorption-incubation procedures (BC+*Lf*). Side view (**B**) and perspective view (**C**) of a CLSM three-dimensional reconstruction of BC+*Lf*$_{24}$ demonstrating that the probiotic does not penetrate through the cellulose network by this preparation method. Green spots (SYTO 9) and red spots (propidium iodide) represent live and dead bacteria, respectively.

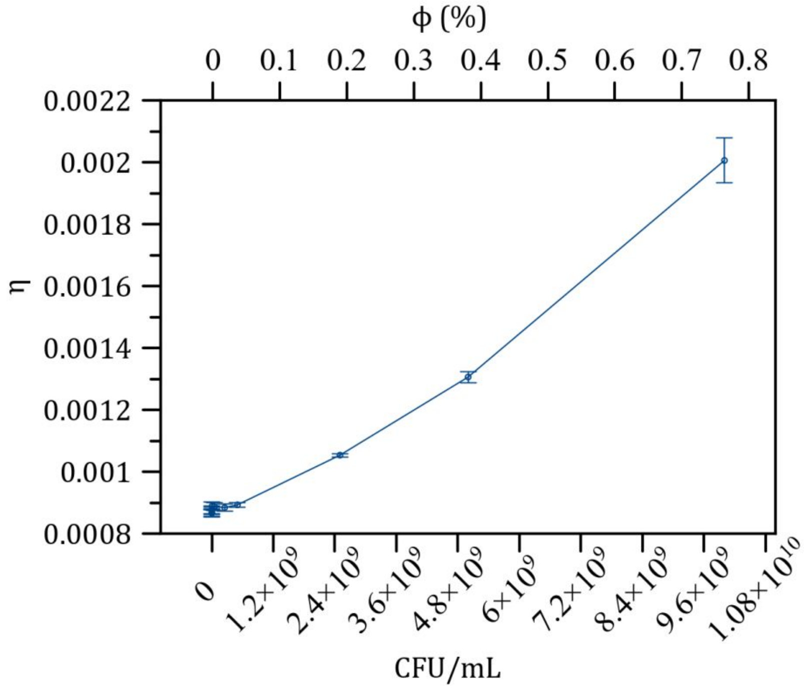

Figure S8. Shear viscosity (η) of a *Lf* suspension with increasing probiotic densities. Data are expressed as mean of triplicates ± SD.
